# Relaxing learned constraints through cathodal tDCS on the left dorsolateral prefrontal cortex

**DOI:** 10.1038/s41598-017-03022-2

**Published:** 2017-06-07

**Authors:** Caroline Di Bernardi Luft, Ioanna Zioga, Michael J. Banissy, Joydeep Bhattacharya

**Affiliations:** 1 Queen Mary University of London, School of Biological and Chemical Sciences, London, E1 4NS United Kingdom; 2 Goldsmiths, University of London, Department of Psychology, London, SE14 6NW United Kingdom

## Abstract

We solve problems by applying previously learned rules. The dorsolateral prefrontal cortex (DLPFC) plays a pivotal role in automating this process of rule induction. Despite its usual efficiency, this process fails when we encounter new problems in which past experience leads to a mental rut. Learned rules could therefore act as constraints which need to be removed in order to change the problem representation for producing the solution. We investigated the possibility of suppressing the DLPFC by transcranial direct current stimulation (tDCS) to facilitate such representational change. Participants solved matchstick arithmetic problems before and after receiving cathodal, anodal or sham tDCS to the left DLPFC. Participants who received cathodal tDCS were more likely to solve the problems that require the maximal relaxation of previously learned constraints than the participants who received anodal or sham tDCS. We conclude that cathodal tDCS over the left DLPFC might facilitate the relaxation of learned constraints, leading to a successful representational change.

## Introduction

We learn and live by experience; our reasoning is biased towards learned rules as we automatically apply learned solutions to identified patterns. This cognitive mechanism is usually efficient, reliable, and almost automatic. There is evidence that the dorsolateral prefrontal cortex (DLPFC) is implicated in readily applying such learned rules with a high degree of automaticity which increases towards its anterior portions^1^. Nonetheless, this automation has a serious cost: it can impair creative problem-solving, especially when the removal of previously learned rules is required^2, 3^. These rules constrain our reasoning space to a number of manageable possibilities subsequently leading to a mental impasse^4^: we get stuck, run out of ideas and are unable to think of a new solution. Such impasses are usually resolved by relaxing previously learned rules or constraints, eventually leading to a successful change in the representation towards the correct solution^5^.

In this study, we explored the impact of modulating DLPFC on creative problem-solving. Given the known role of the prefrontal cortex (PFC) in identifying contextual clues and applying rules that match previously encountered patterns, it might be beneficial to temporarily inhibit the DLPFC in order to facilitate constraint relaxation. Indeed, a seminal study^6^ observed that, compared to healthy controls, patients with lesions in the lateral PFC showed superior performance on problems which require constraint relaxation, suggesting that this brain region is involved in applying well-learned rules or constraints. However, the study also reported that these patients performed worse on problems which demand higher working memory.

In order to temporarily modulate prefrontal activities, we used transcranial direct current stimulation (tDCS). In tDCS, a low direct current (typically <2 mA) is passed from anodal (positive) to cathodal (negative) electrodes positioned on the scalp over target areas^7^. Although the current is too low to trigger an action potential, there is evidence that network modulation of the current triggers an increase in excitability through membrane depolarization in the neurons underneath the anodal, but an inhibition through hyperpolarization under the cathodal electrodes, e.g. refs 8–10. In our study, participants were presented with different types of matchstick arithmetic problems^5^ before and after receiving anodal, cathodal or sham tDCS to the left DLPFC. The matchstick problems varied in terms of the required constraint relaxation for generating the solution, Based on previous work with brain lesion patients^6^, we predicted a benefit of the suppression of the left DLPFC by cathodal tDCS only for problems which require maximal constraint relaxation (problems type C as validated by Knoblich, 1999^5^). We also predicted that cathodal tDCS on the left DLPFC will impair the solution of problems requiring a higher working memory load (in this case B type problems), as it was found in the study with brain lesion patients^6^.

## Methods

### Participants

Sixty healthy individuals (47 females, 13 males) between 18 to 34 years old (*M* = 23.03, *SD* = 4.05 years) took part in this experiment. All participants were right-handed (self-report). Each participant was randomly assigned to one of the three conditions of brain stimulation: anodal (16 females, mean age of 24.05, *SD* = 4.70 years), cathodal (15 females, *M* = 23.3 *SD* = 3.95 years) or sham (16 females, *M* = 22.7, *SD* = 3.19 years). There were no significant age differences between groups (*F*_(2,57)_ = 1.72, *p* = 0.188). All participants were screened for tDCS exclusion criteria, such as neuropsychiatric disorders, history of drug abuse, pregnancy, epilepsy or family history of epilepsy. Knowledge of Roman numerals was a prerequisite for participation; all participants were able to correctly indicate the Roman numerals from I to XX. Written informed consents were obtained from all participants. The study protocol was approved by the ethics committee at Goldsmiths, University of London and performed in accordance with the Declaration of Helsinki.

### Transcranial direct current stimulation (tDCS)

**Figure 1:**
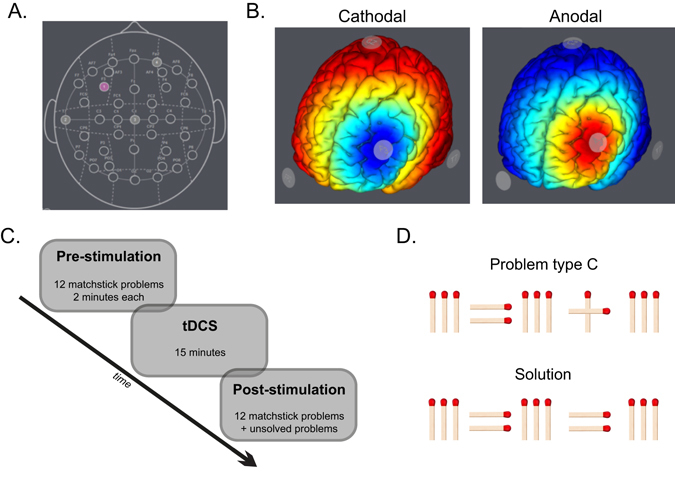
(**A**) An illustration of the placement of electrodes for the tDCS: target electrode (pink) was F3, and return electrodes (light gray) were T7, Cz, and Fp2. (**B**) Simulation of the electric field according to *StimWeaver* (Neuroelectrics, Spain) software based on a realistic head model derived from MR images and the Finite Element Method^11^. (**C**) An illustration of the experimental session. (**D**) An example of a type C matchstick problem.

We used a StarStim® wireless neurostimulator system for active brain stimulation and sham stimulation for 15 minutes. Our target stimulation area was the left dorsolateral prefrontal cortex, which was identified using the F3 scalp location based on the 10–20 system. To focalize the stimulation, we used three return electrodes (T7, Cz, and Fp2) in a triangular location (Fig. 1A). The triangulation placement resulted to only 33% of the current flow to (anodal condition) and from (cathodal condition) the return electrodes, thus restricting the effects of these surrounding electrodes to a very small amount. We simulated the electric field of this montage using the finite element model (FEM)^11^ as implemented in *StimWeaver*, which indicated a focused stimulation of the DLPFC (Fig. 1B). A multisite montage was chosen in order to target the DLPFC as it was found that these montages enable more control over the focality and polarity of the effects^12, 13^. For the active stimulation conditions, a constant current of 1 mA intensity was applied, ramped up (30 seconds) and down (30 seconds) at the start and the end of the stimulation, respectively. For the sham stimulation, current was applied at the ramp periods only. All participants were blind to the type of stimulation condition. The stimulation (anodal, cathodal and sham) was administered immediately after 12 matchstick problems (randomly selected – pre-stimulation).

### Problem-solving task

To investigate the impact of previous experience and relaxation of constraints, we used matchstick arithmetic problems^5, 14^. In a typical matchstick problem, the goal is to correct an incorrect arithmetic statement, expressed in Roman numerals and arithmetic operators (e.g., I, II, +, −) made out of matchsticks, by moving only one matchstick to another place in the equation (see example in Fig. 1D). Participants spontaneously activate their arithmetic knowledge when confronted with such a problem. However, to solve it participants need to relax or even override the learned constraints that are usually applied in arithmetic (e.g. a plus sign cannot turn into an equal sign). The stronger the requirement of constraint relaxation, the harder the problem is to solve. The matchstick problems are categorized into four types depending on the degree of this constraint relaxation^5^:

(i) Type A: This type is solved by moving one matchstick within a specific numeral. For example, the problem IV = III + III is solved by moving the ‘I’ of the ‘IV’ after the ‘V’, i.e. VI = III + III. This is the easiest type as the solver has to override only a value constraint, and it requires decomposition of the values, which are essentially loose chunks.
(ii) Type B: This type is solved by moving one stick from the plus sign to a numeral. For example, I = II + II is solved as I = III − II. Its difficulty is intermediate as the solver has to override both value and operator constraints, and it requires decomposition of the plus sign and the values.
(iii) Type C: This type is solved by rotating the vertical stick of a plus sign changing it into an equal sign, thus creating a tautology. For example, III = III + III is solved as III = III = III. This is the hardest to solve as the individual has to override both the operator and tautology constraints, and it requires decomposition of both the plus and the equal signs. Type C problems are less likely to be solved, but once the participants solve one of them, the subsequent problems are solved nearly every time (the problem is easily identified because of the three equal numerals; see one example in Fig. 1D). Because of this characteristic, comparisons between pre- and post-test are less valid for this sort of problems. Therefore, any differences in the pre-test would affect the post-test heavily and affect our results. To avoid this, we excluded the participants who solved these problems in the pre-test (1 from cathodal, 2 from anodal and 1 from the sham group). Subsequently, we had a similar number of participants in each group (cathodal = 19, anodal = 18, sham = 19).
(iv) Type D: This type is solved by sliding a stick of the symbol ‘X’ to make it a ‘V’. For example, XI = III + III is solved as VI = III + III. Here the solver has to override the value constraint (like Type A), but it requires decomposition of both X and V which are tight chunks.

We presented all four problem types to all participants, and there were 12 problems in the pre- and post-stimulation session (4 type A, 4 type B, 2 type C, and 2 type D). The presentation order of the problems was randomized across participants.

### Procedure

The experiment was conducted in a quiet room. First, participants were given written and oral instructions for the matchstick task. Then, they were asked to solve a practice trial of a Type A problem (IV = III + III), which was laid out using actual matchsticks on the table in front of them. The participants were also presented with the same problem on the screen so they could represent the problem correctly and learn how to enter their solutions. The experimenter demonstrated the correct solution in case they could not find it. During the experiment, each participant was given a table of Roman numerals ranging from 1 to 15. Participants were allowed to use actual matchsticks when searching the solution. The actual matchsticks were used only for demonstration as the experiment was conducted on a computer and the participants had the matchsticks available in case they wished to visualize the problem differently (however, none of the participants made use of this option). As shown in Fig. 1C, during the pre-stimulation session, participants were asked to solve 12 matchstick problems. Except for the practice trial, all matchstick problems were presented on a screen, and participants typed their solution using a keyboard. Participants had up to two minutes per problem. After typing the solution, participants provided ratings on insight (1/0: yes/no insight) and confidence of their solution (1: not at all, 2: quite, 3: very confident). Following the pre-stimulation session, participants received 15 minutes of anodal, cathodal, or sham stimulation, according to the condition to which they were randomly assigned. During the stimulation, all participants completed a set of questionnaires to maintain minimum alertness. This was followed by post-stimulation session in which participants were presented with a new set of 12 different matchstick problems, as well as the problems they did not solve during the pre-stimulation session. In the post-test, the participants received an average of 20 items (SD = 2.1), including a mean of 6.6 for type A (SD = 0.83), 6.8 for type B (SD = 1), 3.3 for type C (SD = 0.71) and 3.3 for type D (SD = 0.73). If the solution was not found within the allocated two minutes, participants were presented with a hint from the computer, highlighting the relevant group of matchsticks including the matchstick which needed to be moved in order to solve the problem. Following the presentation of the hint, participants had up to a minute to solve the problem; subsequently the solution was presented.

### Control measures

Profile of mood states (POMS) questionnaire^15^, a measure of mood, was completed prior to the onset of the electrical stimulation (pre). This allowed to control for potential pre-existing differences in mood states. Further, participants completed the controlled oral association test for measuring verbal fluency, COWAT^16^. Finally, in order to control for inter-group differences in fluid intelligence, we administered the short form of the Raven’s Advanced Progressive Matrices^17^. This short-form is composed of items 1, 4, 8, 11, 15, 18, 21, 23, 25, 30, 31, and 35 of the APM – II Set^17^. The Raven’s and the COWAT were also administered before the tDCS/sham session.

## Results

**Figure 2:**
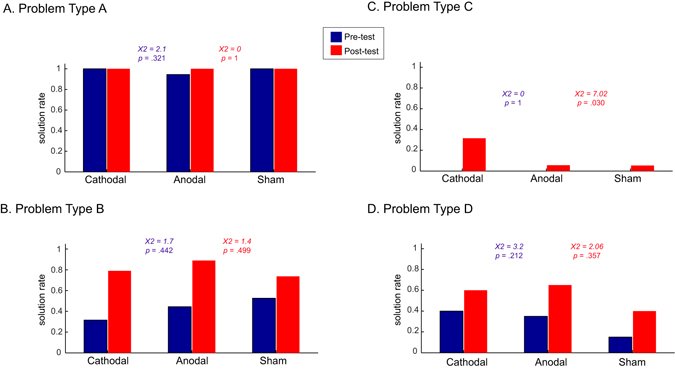
Solution rates (as the percentage of participants who solved the problem) before (blue) and after (red) cathodal, anodal and sham tDCS for problems type A, B, C, and D. Participants who solved problems type C in the pre-test were excluded from the analysis (*n* = 4,/1 from cathodal, 2 from anodal and 1 from the sham group). Solution rate represents the proportion of participants within each stimulation group who managed to solve the problem correctly in the corresponding session (pre- and post-test). We tested the difference in the proportion of participants solving the problem between groups using chi-square. The numbers in blue are the results for the differences between groups in the pre-test and in red for the post-test. The proportion of participants who successfully solved the problems was significantly different between groups only for problems type C in the post-test.

We calculated three performance indices for each type of problem: (1) *solution rate*, as the proportion of participants who solved the problem at least once (pre- and post-test separately); (2) *success index*, as the proportion of participants who solved the problem for the first time at least once (excluding participants who solved the problem in the pre-test); and (3) *relax index*, as the proportion of correct solutions after having solved the problem for the first time. The latter two measures (success index and relax index) followed the procedures adopted by Reverberi *et al*.^6^. The difference in the solution rates between pre- and post-test for each problem type is presented in Fig. 2.

The results showed that problems type A were the easiest (ceiling effect) and were solved in both pre- and post-test. The other problems (B, C and D) were more likely to be solved in the post-test. We had a clear and specific prediction that the group which received cathodal stimulation over the left DLPFC would be more likely to solve the most difficult matchstick problems (type C) than the groups receiving anodal or sham tDCS over the same area. In order to test whether cathodal stimulation affected the solution rates for problem type C, as in the previous study^6^, we used chi-square tests to compare the proportion of participants solving the problem before and after the stimulation between groups (one chi-square for the pre- and another for post-test). We found that in the post-test there was a higher proportion of participants (32%) who could solve type C problems following cathodal tDCS as hypothesised (*X* ^*2*^ = 7.02, *p* = 0.030) than following anodal (5%) and sham (5%). None of the other problems or conditions were significantly different between groups, demonstrating that the stimulation effects were, as predicted, specific to type C problems.

We compared the success index in the post-test between groups using chi-square. The success index for type A problems could not be computed since all participants solved it during the pre-test. The success index for problems ﻿type B did not differ between the stimulation groups in the post-test (*X* ^*2*^ = 1.68, *p* = 0.433). The success index for problem﻿s type B type was only slightly higher for anodal tDCS group (80% within the group) compared to cathodal (76.9%) and sham (55.6%). For problems type C, the difference was significant (*X* ^*2*^ = 7.02, *p* = 0.030). We predicted that this difference was driven by the higher number of participants solving problems type C after cathodal rather than sham and anodal tDCS. Planned contrasts (*2* × *2 chi-square)* revealed that cathodal tDCS led to significantly larger success index compared to anodal tDCS (*X* ^*2*^ = 4.08, *p* = 0.043), and to sham (*X* ^*2*^ = 4.38, *p* = 0.036), but no difference between anodal and sham (*X* ^*2*^ = 0.002, *p* = 0.969). For problems type D, there was a slightly higher success index for anodal (46.2%) compared to cathodal (33.3%) and sham (29.4%), but this difference was not statistically significant (*X* ^*2*^ = 0.94, *p* = 0.625). Importantly, we also contrasted cathodal vs. sham and cathodal vs. anodal (and anodal vs. sham) for all problems in both pre- and post-test, and observed that all contrasts were non-significant (*p* > 0.2), supporting the hypothesis of specific effects for cathodal tDCS on problems type C.

We also calculated the improvement as the percentage of solved problems in the post-test minus the same percentage in the pre-test for each participant. There was an improvement from pre- to post-test for type B problems (*Wilcoxon*’*s Z* = *−*4.2, *p* < 0.001), type C (*Z* = *−*2.83, *p* = 0.005), and problems type D (*Z* = *−*3.87, *p* < 0.001), but not for problem type A (*Z* = *−*1, *p* = 0.317). Comparing the improvement score (post-test minus pre-test) between the three groups revealed that the groups differed only for problems type C (*Kruskal Wallis X* ^*2*^ = 7.01, *p* = 0.030), but not for the other types of problems (type A: *X* ^*2*^ = 1.57, *p* = 0.456; type B: *X* ^*2*^ = 3.48, *p* = 0.176; type D: *X* ^*2*^ = 0.44, *p* = 0.801). Planned contrasts showed that the improvement for problems type C was higher for cathodal than for sham (*Mann-Whitney U* = 139, *p* = 0.018) and anodal tDCS (*Mann-Whitney U* = 144, *p* = 0.046).

**Figure 3:**
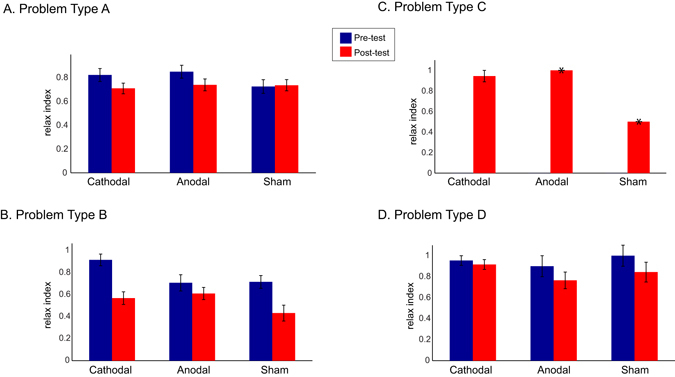
Relax index (as the percentage of time the problem was solved after having solved that problem once) before (blue) and after (red) cathodal, anodal and sham tDCS for problem types A, B, C, and D. Relax index is the proportion of correct solutions after the problem was solved for the first time. The error bars represent +/−1 S.E.M. Note that for the problem type C there was only one participant in the post-test anodal and one in the sham conditions, which prevented the use of error bars (*).

Figure 3 shows the relax index for the four problem types. Replicating previous findings^5, 6^, problems type C showed the largest relax index, as they become particularly easy once they are solved for the first time, that is after the constraints are relaxed. We compared the relax indices for the participants who solved the problems between the three groups using the non-parametric *Kruskal-Wallis test*, which revealed no significant differences between the groups in either pre- or post-test relax indices (*p* > 0.05), except for the pre-test on problems type B which was higher for the cathodal group (*Kruskal-Wallis X* ^*2*^ = 9.9, *p* = 0.007). This difference became non-significant in the post-test (*Kruskal-Wallis X* ^*2*^ = 2.5, *p* = 0.286). Across groups, we found a significant decrease in the relax score from pre- to post-test for problems type B only (*Z* = *−*2.53, *p* = *0*.*012*) and observed a similar trend for problems type A (*Z* = *−*1.85, *p* = *0*.*065*) and type D (*Z* = *−*1.81, *p* = *0*.*071*). Comparing the relax index gain score (post-test minus pre-test) between groups, we found that the groups differed for problems type B only (*X* ^*2*^ = 6.86, *p* = 0.032), but not for the other types (*p* > 0.1). Post-hoc contrasts revealed that the decrease in performance was higher after cathodal compared to anodal tDCS (*U* = 18.5, *p* = 0.010) and sham (*U* = 26, *p* = 0.047), whereas there was no difference between anodal and sham (*U* = 104, *p* = 0.381). Therefore, the cathodal group seems to have decreased their efficiency to solve problems type B after the stimulation, but that difference could be due to a higher pre-test relax index.

Next, we investigated the impact of stimulation on response times. Comparing the response times between participants in the pre- and post-test separately for each problem type (except C because they were not solved in the pre-test), we found no significant differences between the groups (*p* > 0.05). Pairwise contrasts (pre- vs. post-test) revealed that participants became faster in the post-test, but only for type A (*Z* = *−*2.71, *p* = *0*.*007*), and only in the sham group (*Z* = *−*2.25, *p* = 0.024). However, contrasts between groups revealed no significant differences. Neither we found significant differences between the groups in terms of response time gain score between pre- and post-test, suggesting no significant interaction between stimulation condition and solution times (*X* ^*2*^ = 0.146, *p* = 0.930).

Finally, we compared self-reported insight as the percentage of insight vs. non-insight solutions reported by the participants. First, we analysed the proportion of participants who reported solving a problem with insight when correctly solving it for the first time. For type A problems, we observed that 53.6% of the participants reported solving these with insight when solving for the first time. This percentage was slightly higher for type B (55.3%) and type C (62.5%) problems. The lowest percentage of insight was found for problems type D (48.5%). We compared the distribution between stimulation conditions (one chi-square per problem type) and found no significant differences in any of the problem types (*p* > 0.4). We also assessed the proportion of insight solutions per participant (considering all trials rather than just the first solution) in the pre- and post-test and compared them between these conditions using the Wilcoxon signed-rank test. On average, 40% (SD = 38) of the type A problems were solved with self-reported insight compared to 45% (SD = 31) in the post-test, but this difference was not statistically significant (Wilcoxon’s *Z* = −*1*.*11*, *p* = 0.267). There was also no difference for problems type B, which were on average solved with insight about 38% (SD = 35) of the time in the pre- and 33% (SD = 29) in the post-test (*Z* = −*0*.*4*, *p* = 0.672). The same was observed for D type problems (*Z* = −*1*.*03*, *p* = 0.303), with an average of 47% (SD = 39) in the pre-test and 53% (SD = 37) in the post-test. Problems type C were not rated for insight in the pre-test because they were not solved, but on average in the post-test, these problems were solved with insight 52% of the time (SD = 35). No differences were observed between the groups in either pre- or post-test in the number of solutions perceived as an insight vs. non-insight by the participants (*p* > 0.05).

### Further control analyses

In order to control for possible pre-existent differences between the three groups, we collected information regarding fluid intelligence, mood states, personality, and verbal fluency *(*see *Methods*). Groups did not differ in any of these measures (*X* ^*2*^ < 2, *p* > 0.2 in all comparisons).

Further, we compared the number of trials presented in the post-test for all trials and for each trial separately using a one-way ANOVA with the number of trials as the dependent variable and the stimulation group as a factor (independent variable). There was no significant difference between groups in relation to the number of trials (*F*(2,55) = 0.529, *p* = 0.592) or for problem types A, B, C and D (*F* < 1, *p* > 0.4).

## Discussion

In this study, we showed that cathodal stimulation over the left DLPFC could help break mental constraints learned from experience. Using tDCS, we were able to replicate the results of a seminal brain lesion study^6^ which found that patients with lesions on the DLPFC were better at solving problems in which the solution depended almost exclusively on relaxing mental constraints (e.g., IV = IV + IV, solution: IV = IV = IV). Solving such problems depends on relaxing both operator (e.g., turn a + into =) and tautology (the correct statement contains two equal signs) constraints. In these problems it is also necessary to decompose chunks, that is to realise the individual parts which form one element^5^. We also found that cathodal tDCS was associated with a decrease in relax index for type B problems, which require the relaxation of the value and operator constraints. In the aforementioned study^6^, it was also observed that controls outperformed patients in problems type B. Altogether, our findings suggest that it might be possible to temporarily reproduce cognitive patterns observed in brain lesion patients by multi-site cathodal tDCS.

A previous study using tDCS has demonstrated that cathodal stimulation over the left DLPFC can influence creativity^18^. In particular, cathodal tDCS over the left DLPFC was associated with better performance in an uncommon uses tasks in which the participants had to come up with different uses for objects. Another study^19^ observed the opposite: anodal tDCS over the left and cathodal over the right DLPFC improved creative problem solving in a remote word association task. A previous study^20^ using matchstick tasks observed that anodal on the right temporal lobe coupled with cathodal over the left was associated with breaking the mental set in difficult matchstick problems, although different stimulation parameters were used (e.g. intensity of stimulation, which has been shown to change shifts in cortical excitability^21^). Due to stimulation montage and intensities of stimulation used in these studies, it is difficult to ascertain specific shifts in cortical excitability that contributed to the observed changes. To the best of our knowledge, this is the first study applying multisite tDCS at a 1 mA intensity to promote a more focal stimulation in order to understand whether inhibiting the left DLPFC could contribute to relaxing learned constraints.

Our findings support the idea that certain cognitive functions, especially those required in creative problem solving, could benefit from inhibition rather than excitation of the PFC^2^. We hypothesize that the inhibition of the left DLPFC facilitates constraint relaxation by reducing the strength of automatic processes implemented to solve problems triggered by contextual clues^1^. According to *the matched filter hypothesis*, the PFC functions as a filter and biases competing representations in order to optimize sensory processing and decision making^18^; the lateral PFC searches for matches and applies the most likely solution. This is an efficient and reliable strategy for solving well-known problems similar to what we have encountered in the past, but not for those problems which require a representational change. Our results suggest that it might be possible to temporarily inhibit such filters, which may or may not help finding the desired, and unusual, optimal solution. We speculate that constraint relaxation depends on such deactivation of the lateral PFC, and that the constraints act as filters.

We also observed a decrease in performance for type B problems. According to Reverberi *et al*.^6^, this type has twice as many possible matchstick moves (on average 10.2, compared to 5.7 and 5.5 for types A and C, respectively), creating a higher computational load as the participant needs to search over a larger solution space. This would require the participants to keep the attempted (and failed) solutions in their working memory, which is also a function of the DLPFC (for a review: ref. 22). Therefore, inhibiting the DLPFC would impair performance on these problems. This could also explain why participants with high working memory capacity show worse performance at type C problems and better at type B^23^ problems. These findings highlight the importance of a dynamic and balanced activity over different brain areas once the same tDCS protocol can improve and impair performance on problems that are, on the surface, similar. Even subtle differences in the problem structure (e.g., type of constraint, chunks, and solution space) seem to have an impact on the effects of such protocols, which is a big obstacle for more generalised claims such as that tDCS can improve creativity. We conclude that cathodal tDCS over the left DLPFC can increase the likelihood of solving matchstick problems requiring the inhibition of learned constraints and decrease the likelihood of solving matchstick problems with a higher working memory load.

Nevertheless, our findings must be interpreted with caution. It is important to notice that we observed a lower rate of solutions for problems type C compared to previous studies adopting the same experimental paradigm^6, 20, 24^. This might be due to two reasons. First, we gave the participants only 2 minutes to solve each problem compared to 3 minutes plus an extra minute with a hint in the brain lesion study^6^ or 6 minutes in a brain stimulation study^20^. The second reason might be our larger number of problems (12 in the pre-test followed by another 12 plus the non-solved ones). Solving other matchstick problems which require different representational changes was found to make the participants less likely to solve problems requiring stronger constraint relaxation, by making them fixated on the wrong solution^24^. A previous study^24^ observed, across three experiments, that solving problems requiring a representational change such as chunk decomposition (e.g. VI = VI + I; solution: VI = VII − I) inhibited the solution of difficult insight problems such as problems type C. In fact, the authors found that less than 10% of the participants were able to solve problems type C in less than 2 minutes after inducing a mental set through asking the participants to solve other matchstick problems (types A and B). Therefore, a mental set could have been induced by our large set of problems. This is connected to our second limitation: we excluded the small number of participants who successfully solved the type C problems in the pre-test because these problems become obvious to solve once the constraints are relaxed for the first time. If we consider the possibility that solving the other items induced a mental set, we could assume that these participants were less likely to develop a mental set by solving other problems. Therefore, our results can only be generalised to participants who are more likely to show a mental set when exposed to other problems. We do not know whether the effects of cathodal tDCS over the left DLPFC are specific to participants who are more prone to show a mental set or not, and this hypothesis remains to be tested. Finally, another limitation concerns the effects of the anodal tDCS over the left DLPFC on solving type C problems. The fact that we have not found any impairment associated with this protocol does not mean anodal tDCS cannot potentially *increase* the mental set. Since not many people are able to solve this type of problems, it is possible that the mental set could get worse but because of this floor effect we were not able to observe it. We suggest that future studies analyse the effects of anodal and cathodal tDCS over the DLPFC on other problems which can induce a mental set without causing a floor effect.

In summary, we demonstrated that suppressing the left DLPFC in healthy controls facilitated representational change in solving difficult trials of a novel problem by relaxing previously learned constraints in a similar counterintuitive fashion as found in patients with prefrontal lesions.
